# IL-3 but not monomeric IgE regulates FcεRI levels and cell survival in primary human basophils

**DOI:** 10.1038/s41419-018-0526-9

**Published:** 2018-05-03

**Authors:** Fabian Zellweger, Patrick Buschor, Gabriel Hobi, Daniel Brigger, Clemens Andreas Dahinden, Peter Matthias Villiger, Alexander Eggel

**Affiliations:** 1 Department for BioMedical Research, University of Bern, Bern, Switzerland; 2 Department of Rheumatology, Immunology and Allergology, University Hospital Bern, Bern, Switzerland; 3 former Institute of Immunology, University of Bern, Bern, Switzerland

## Abstract

Binding of allergen-specific IgE to its primary receptor FcεRI on basophils and mast cells represents a central event in the development of allergic diseases. The high-affinity interaction between IgE and FcεRI results in permanent sensitization of these allergic effector cells and critically regulates their release of pro-inflammatory mediators upon IgE cross-linking by allergens. In addition, binding of monomeric IgE has been reported to actively regulate FcεRI surface levels and promote survival of mast cells in the absence of allergen through the induction of autocrine cytokine secretion including interleukin-3 (IL-3). As basophils and mast cells share many biological commonalities we sought to assess the role of monomeric IgE binding and IL-3 signaling in FcεRI regulation and cell survival of primary human basophils. FcεRI cell surface levels and survival of isolated blood basophils were assessed upon addition of monomeric IgE or physiologic removal of endogenous cell-bound IgE with a disruptive IgE inhibitor by flow cytometry. We further determined basophil cell numbers in both low and high serum IgE blood donors and mice that are either sufficient or deficient for FcεRI. Ultimately, we investigated the effect of IL-3 on basophil surface FcεRI levels by protein and gene expression analysis. Surface levels of FcεRI were passively stabilized but not actively upregulated in the presence of monomeric IgE. In contrast to previous observations with mast cells, monomeric IgE binding did not enhance basophil survival. Interestingly, we found that IL-3 transcriptionally regulates surface levels of FcεRI in human primary basophils. Our data suggest that IL-3 but not monomeric IgE regulates FcεRI expression and cell survival in primary human basophils. Thus, blocking of IL-3 signaling in allergic effector cells might represent an interesting approach to diminish surface FcεRI levels and to prevent prolonged cell survival in allergic inflammation.

## Introduction

Binding of allergen-specific immunoglobulin E (IgE) to its high-affinity receptor FcεRI expressed on basophils and mast cells is a central step in the induction of allergic hypersensitivity reactions^1^. On these cells, FcεRI is expressed as a hetero-tetramer^2^. IgE binding occurs asymmetrically via two binding sites in the extracellular part of the FcεRI α-subunit (FcεRIα)^3^. The membrane tetra-spanning β-chain (FcεRIβ), and the two identical disulfide-linked γ-chains (FcεRIγ) are not involved in the IgE interaction but are essential for receptor maturation, receptor surface transport, and the propagation of FcεRI-mediated signaling pathways^4^. Owing to the high-affinity interaction of IgE:FcεRI complexes basophils and mast cells are permanently sensitized with IgE and are thus ready to immediately respond to allergen challenge. Antigen-induced co-aggregation of receptor-bound IgE stimulates the release of pre-stored and *de novo* synthesized mediators that induce classical allergy symptoms including bronchoconstriction, vasodilatation, and increased mucus production^5^.

In 1978, researchers have observed that serum IgE levels of atopic as well as non-atopic subjects positively correlate with surface FcεRI levels on primary human basophils^6^. These findings have sparked the hypothesis that IgE might be involved in the regulation of FcεRI expression on allergic effector cells. More recently, *in vivo* studies using the therapeutic monoclonal anti-IgE antibody omalizumab have confirmed that reducing serum IgE level in atopic patients goes along with decreased FcεRI levels on human basophils and mast cells^7–9^. In addition, various studies reported that monomeric IgE is directly involved in the regulation of surface FcεRI expression in murine bone marrow-derived mast cells and human umbilical cord blood-derived mast cells^10–13^. Binding of monomeric IgE to FcεRI has furthermore been described to promote survival of mBMMC in the absence of allergen^14–17^. Interestingly, the mechanisms underlying IgE-mediated cell survival suggested in these studies greatly differed. Although one study attributed prolonged mBMMC survival to the induction of autocrine cytokine secretion and elevated expression of the antiapoptotic Bcl-2 family member Bcl-X_L_^15^, the other  study specifically excluded both of these possibilities^14^. Subsequent investigations have suggested that particularly the induction of autocrine interleukin-3 (IL-3) secretion is responsible for the enhanced mast cell survival upon monomeric IgE binding^18^. How the monomeric interaction between IgE and FcεRI can induce intracellular signaling cascades in mast cells remained obscure until it was found that there are so-called highly cytokinergic (HC) IgE clones^19,20^. Most of these HC IgE clones exhibit polyreactivity to autoantigens or display self-reactivity^21–23^. They may therefore induce FcεRI aggregation in the absence of allergen, which could lead to the induction of cytokine production including IL-3^24^. For basophils and mast cells, IL-3 is a well-characterized proliferation and survival factor^25–27^. Thus, the enhanced survival that has initially been attributed to monomeric IgE binding might be owing to IgE aggregation on the cell surface in the absence of allergen. Whether HC IgE is present *in vivo* and if it has a role in the regulation of mast cell survival remains elusive.

Despite controversial findings with mast cells, we aimed to investigate the effect of monomeric IgE binding and IL-3 signaling on FcεRI regulation and cell survival of human primary blood basophils in this study. We have recently generated a novel disruptive anti-IgE DARPin^®^ protein (bi53_79)^28–30^, that we used as a tool to actively remove IgE from the cell surface under physiological conditions. Our results demonstrate that removal of IgE leads to loss of FcεRI on the cell surface, whereas FcεRI-bound IgE stabilizes receptor levels. However, the presence and amount of monomeric IgE did not influence the survival of *in vitro* cultured primary human basophils or change the number of measured blood basophil numbers *in vivo*. Further, our results provide strong evidence that IL-3 signaling but not monomeric IgE binding regulates FcεRI levels on the basophil cell surface.

## Results

### IgE stablilizes FcεRI on human primary basophils

**Fig. 1:**
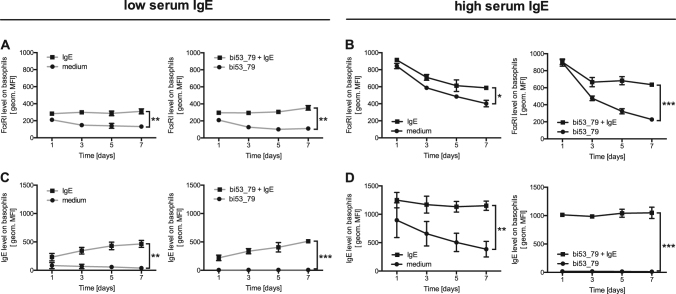
Regulation of surface FcεRI levels by monomeric IgE. Kinetics of FcεRI **a** and **b** and IgE levels **c** and **d** on isolated primary human basophils from low ( ≤ 32 kU/L; gray lines) and high ( > 32 kU/L; black lines) serum IgE donors over a 7-day time period. Cells were incubated either in medium alone, with exogenous recombinant human IgE (100 nM), with the disruptive anti-IgE DARPin^®^ protein bi53_79 (5 µM) or with the disruptive anti-IgE DARPin^®^ protein bi53_79 and subsequently after washing with exogenous recombinant human Sus11-IgE. FcεRI and IgE levels were measured by flow cytometry and displayed as geometric mean fluorescence intensity. Statistical significance was calculated by Student’s *t*-test at day 7 and data are shown as mean ± SEM. *p *<* 0.05, **p *<* 0.01, ***p *<* 0.001, *n* = 3

To investigate the effect of IgE on the surface expression of FcεRI we isolated primary human blood basophils from donors with low ( ≤ 32 kU/L) and high serum IgE levels ( > 32 kU/L). Basophils were either incubated with medium, exogenous recombinant human IgE, bi53_79 to remove endogenous IgE or with bi53_79 prior to the addition of exogenous recombinant human IgE. The FcεRI and IgE cell surface levels were determined over a period of 7 days. Compared with incubation in medium the addition of exogenous IgE reduced the loss of FcεRI on basophils from both low (Fig. 1a) and high serum IgE (Fig. 1b) donors. In line with this observation, the removal of endogenous IgE with bi53_79 resulted in an accelerated loss of FcεRI in both groups. Stripping and subsequent reloading with exogenous IgE also led to a reduction in receptor loss. Basophils from low IgE donors treated with exogenous IgE showed slightly increased IgE surface levels (Fig. 1c), whereas those on basophils from high serum IgE-remained stable (Fig. 1d). In agreement with previous studies^31^, these experiments demonstrate that the addition of monomeric IgE reduces the loss FcεRI on basophils from both low and high serum IgE level donors.

### Monomeric IgE binding does not influence survival of human blood basophils

**Fig. 2:**
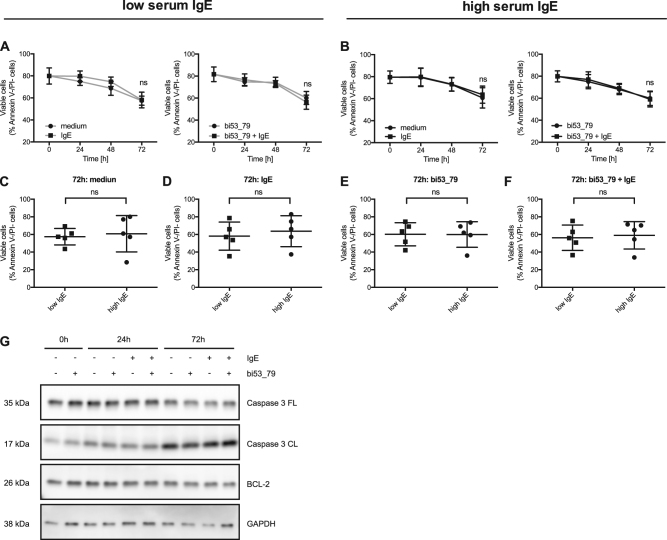
Survival of primary human basophils in the presence or absence of monomeric IgE. Isolated primary human basophils from low ( ≤ 32 kU/L) and high ( > 32 kU/L) serum IgE donors were analyzed for cell viability over a 72-hour time period. **a** and **b** Cells were incubated either in medium alone, with exogenous recombinant human IgE (100 nM), with the disruptive anti-IgE DARPin^®^ protein bi53_79 (5 µM) or with the disruptive anti-IgE DARPin^®^ protein bi53_79 and exogenous recombinant human Sus11-IgE in combination. Comparison of cell viability between basophils from low and high serum IgE blood donors at 72 h incubation in medium **c**, IgE **d**, bi53_79 **e** or bi53_79 and IgE **f**. A comparative western blot analysis of pro- (i.e., cleaved caspase-3) and antiapoptotic proteins (i.e. BCL-2) in primary human basophils from three blood donors that were cultured for 0, 24 and 72 h in medium, IgE, bi53_79 or bi53_79 and IgE **g**. GAPDH serves as loading control. Statistical significance was calculated by Student’s *t*-test at 72 h and data are shown as mean ± SEM, *n* = 5

We next assessed whether IgE binding might enhance survival of primary human basophils as previously described for mast cells^14,15^. For this purpose, we compared the *ex vivo* survival of isolated human basophils and assessed the effect of IgE removal with bi53_79 versus addition of exogenous IgE. Surprisingly, we did not observe a difference in cell survival over the period of 72 h (Fig. 2a,b). Neither endogenous cell-bound IgE nor experimental removal or addition of recombinant IgE had a significant effect on cell viability *ex vivo*. Along this line, we could not observe a difference in survival between basophils isolated from low or high serum IgE donors after 72 h of culture (Fig. 2c–f). In addition, we performed western blot analysis with basophils of three donors to further assess intracellular pro- and antiapoptotic signaling pathways on a molecular level. In line with the cell viability data measured by flow cytometry we did not observe any significant difference in pro-apoptotic caspase-3 cleavage nor an induction of antiapoptotic molecule Bcl-2 upon addition of monomeric IgE (Fig. 2g). As a control, we generated bone marrow-derived mast cells (BMMCs) from mice that lack murine FcεRIα but transgenically express functional human FcεRIα (huFcεRIα^tg^)^32^. Addition of murine IL-3 but not monomeric IgE prolonged the survival of these cells as assessed by flow cytometry and western blot (Fig. S1). These data strongly indicate that the monomeric IgE clones used in this study do not enhance the survival of human primary basophils or BMMCs from huFcεRIα^tg^ mice *ex vivo*.

### IgE levels in humans or lack of FcεRIα in mice does not alter blood basophil counts

**Fig. 3:**
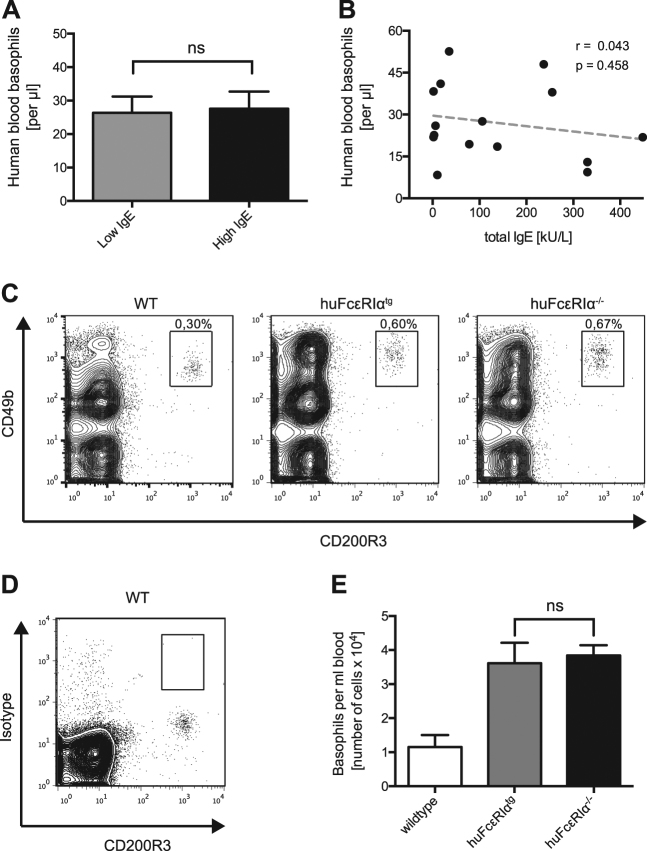
In vivo basophil counts in human low and high serum IgE donors and mice that are sufficient or deficient for FcεRI. Comparison of basophil cell counts in blood from low ( ≤ 32 kU/L, *n* = 6, gray bars) versus high ( > 32 kU/L, *n* = 9, black bars) serum IgE donors **a**. **b** Correlation between blood basophil cell counts and total serum IgE in different blood donors (*n* = 15). **c** Representative flow cytometry measurement of CD49^+^/ CD200R3^+^ blood basophils in wildtype, huFcεRIα^tg^ and huFcεRIα^−/−^ mice. **d** Isotype staining control for CD49b. **e** Absolute quantification of blood basophil cell counts in wildtype (*n* = 6), huFcεRIα^tg^ (*n* = 4), and huFcεRIα^−/−^ (*n* = 8) mice. Data are shown as mean values ± SEM

To assess whether the presence of high IgE levels regulates circulating basophil numbers *in vivo* we quantified blood basophil numbers of human individuals with low ( ≤ 32 kU/L) or high ( > 32 kU/L) IgE serum levels by flow cytometry. There was no significant difference in the blood basophil counts between these two groups (Fig. 3a). In addition, we did not find any correlation of IgE serum levels with blood basophil counts (Fig. 3b). To investigate whether FcεRI signaling is involved in the survival of murine basophils *in vivo* we quantified blood basophil numbers in mice lacking the murine FcεRIα but that are transgenic for the human FcεRIα (huFcεRIα^tg^) by flow cytometry. These cell numbers were compared to the basophil counts of littermates lacking both the murine and the human FcεRIα (huFcεRIα^−/−^) (Fig. 3c,d). As an additional reference, we also included wildtype C57BL/6 (WT). It is known that murine IgE has the ability to bind to human FcεRIα^33^ and that human FcεRIα signaling is functional in huFcεRIα^tg^ mice^32^. In addition to our observation that monomeric IgE did not affect survival of human blood basophils *ex vivo*, we did not detect any changes in blood basophil numbers between huFcεRIα^tg^ and huFcεRIα^−/−^ mice (Fig. 3e). The increased basophil counts that could be observed between WT and transgenic mice is likely owing to differences in the genetic background. Our finding that the lack of FcεRIα does not affect absolute basophil numbers in mice is in line with previous findings that show no difference in survival of basophils from wildtype and IgE knockout mice^34^.

### IL-3 increases FcεRI levels on human basophils

**Fig. 4:**
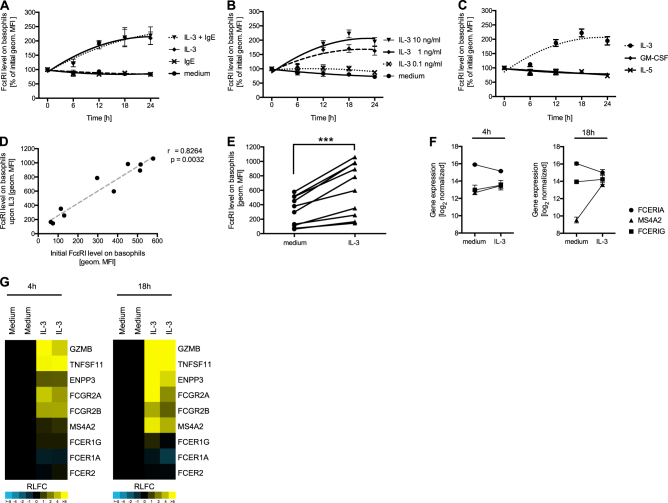
IL-3-mediated increase of surface FcεRI levels. Isolated primary human basophils were incubated for 24 h with medium, IL-3 (10 ng/ml), Sus11-IgE (100 nM) or IL-3 and IgE in combination **a**, different concentrations of IL-3 (0.1−10 ng/ml) **b**, IL-3 (10 ng/ml), GM-CSF (10 ng/ml) or IL-5 (10 ng/ml) **c**. FcεRI surface levels were determined by flow cytometry and the percentage change compared to initial geometric mean fluorescence intensities are shown. The correlation between the initial FcεRI level with the FcεRI level after 24-hour incubation with IL-3 (10 ng/ml) on the surface of basophils isolated from different donors (*n* = 10) is displayed **d**. FcεRI levels on the surface of isolated basophils from different donors (*n* = 10) after 24-hour incubation with either medium or IL-3 (10 ng/ml) are compared **e**. Normalized and log2-transformed gene expression results for FCERIA (FcεRIα), MS4A2 (FcεRIβ), and FCERIG (FcεRIγ) are shown from two independent arrays using pooled basophil RNA of three individual donors after incubation with medium or IL-3 for 4 or 18 h **f**. The relative log2 fold change (RLFC) of IL-3-treated basophils is shown as a heat-map for the indicated genes **g**. Data are shown as mean values ± SEM

IL-3 is a well-characterized pro-survival cytokine for primary human basophils, which is commonly used to extend their viability *ex vivo*^27^. As we did not observe an active upregulation of FcεRI levels on basophils by monomeric IgE, we asked whether IL-3 might be involved in receptor regulation. Thus, we performed cell culture assays with isolated primary human basophils in the presence or absence of recombinant human IL-3. Upon incubation of the cells with IL-3 we detected a time-and dose-dependent increase of FcεRI receptor levels by flow cytometry (Fig. 4a,b). Other cytokines that signal through the same receptor family on primary human basophils, such as IL-5 and granulocyte–macrophage colony-stimulating factor (GM-CSF), did not induce changes in FcεRI surface levels (Fig. 4c). These data indicate that FcεRI levels are specifically regulated through IL-3 receptor signaling. Interestingly, IL-3 mediated upregulation of surface FcεRI showed a strong correlation to the initial donor-specific receptor level (Fig. 4d). In other words, donors with high initial FcεRI levels displayed an increased accumulation of new receptor compared with donors with low initial FcεRI levels. To gain further insight into this IL-3-dependent mechanism of receptor regulation we performed gene expression analysis. Isolated primary blood basophils were incubated in medium or medium containing IL-3 for 4 or 18 h. Basophil RNA was extracted and subsequently used for whole-genome expression measurements. Surprisingly, bioinformatic analysis revealed that transcription of FCERIA is slightly downregulated upon incubation of the cells with IL-3. However, MS4A2 the gene encoding the FcεRIβ was highly upregulated after 18 h in presence of IL-3 (Fig. 4f,g). Other genes including GZMB^35^, TNFSF11^36^, ENPP3^37^, FCGR2A, and FCGR2B^38^ are known to be transcriptionally upregulated by IL-3 and are shown as controls. It has previously been reported that FcεRIβ is an amplifier of FcεRI cell surface expression^2,39,40^. It does so by promoting maturation and trafficking of the associated FcεRIα. In addition, FcεRIβ might stabilize the cell surface expressed FcεRI complex. We also found a moderate transcriptional upregulation of FCERIG, the gene encoding the FcεRIγ upon IL-3 incubation. Previous studies have demonstrated that cells lacking FcεRIγ transcripts do not express surface FcεRI. Taken together our data suggest that the IL-3-mediated increase in FcεRI levels on primary human basophils is owing to transcriptional upregulation of FcεRIβ and FcεRIγ but not FcεRIα.

### IL-3 induced FcεRI binds IgE and is functionally active

**Fig. 5:**
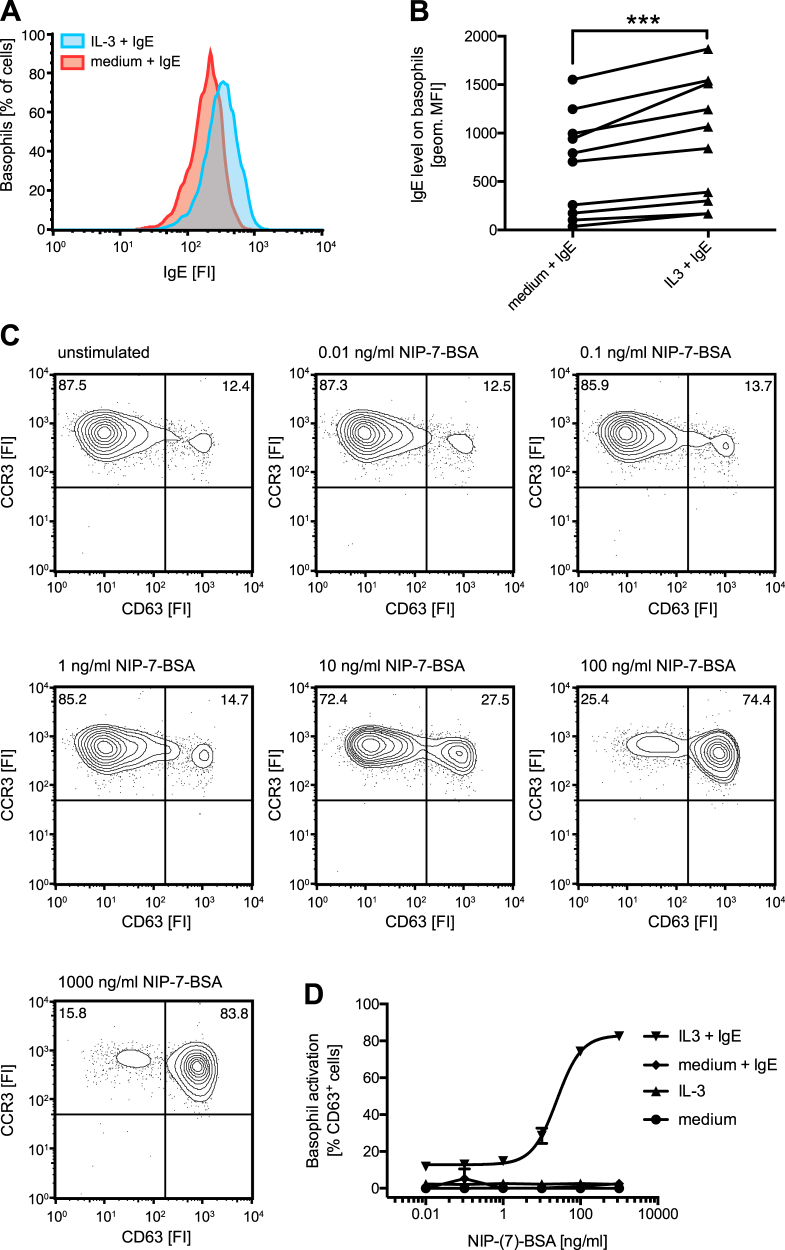
IL-3 induced FcεRI on human basophils is functionally active. Isolated primary human basophils were incubated with medium or IL-3 (10 ng/ml) overnight and sensitized with recombinant monomeric human NIP-specific JW8-IgE (100 nM) for 30 minutes. Surface IgE levels were determined by flow cytometry. **a** A representative histogram indicating the increase in fluorescence intensity (FI) upon incubation of IL-3 primed cells with IgE is shown. IgE levels on the surface of isolated basophils from different donors (*n* = 10) after overnight incubation with medium or IL-3 (10 ng/ml) and subsequent addition of IgE (100 nM) are compared **b**. IL-3 primed JW8-IgE-sensitized basophils were stimulated with increasing concentrations of NIP-7-BSA antigen. Activation was determined by measuring CD63 surface expression using flow cytometry. Representative dot plots are shown **c**. Quantification of antigen-challenged basophils from different donors that were incubated with or without IL-3 overnight and sensitized with or without JW8-IgE for 30 minutes (*n* = 3). Data are shown as mean values ± SEM

Next, we assessed whether IL-3-induced FcεRI binds IgE and whether it is functional in terms of IgE-dependent antigen stimulation. To determine whether the newly displayed receptor is capable of binding IgE, isolated primary human basophils were cultured in the presence or absence of IL-3 for 18 h and subsequently incubated with recombinant human IgE. Basophils of all donors showed increased IgE levels in the presence of IL-3 as assessed by flow cytometry (Fig. 5a,b). To investigate the functionality of the newly displayed receptor we used recombinant 4-Hydroxy-3-iodo-5-nitrophenylacetyl (NIP)-specific human IgE in these basophil cultures. Subsequent stimulation with NIP-(7)-BSA antigen resulted in a dose-dependent activation exclusively of those cells that were incubated in IL-3 and NIP-specific human IgE (Fig. 5c,d). In the absence of IL-3, where no FcεRI increase occurs, basophils could not be activated upon antigen challenge. These results demonstrate that IL-3-induced FcεRI on the surface of primary human basophils is functional and that IL-3 signaling under inflammatory conditions may increase basophil sensitivity and reactivity.

## Discussion

In this study, we have evaluated the effect of monomeric IgE and IL-3 on FcεRI regulation and cell survival in primary human basophils. To carefully dissect the role of IgE in this context, we took advantage of the disruptive anti-IgE DARPin^®^ protein bi53_79, which removes endogenous receptor-bound IgE from the basophil cell surface under physiological conditions. Also, we additionally separated basophil donors into two groups based on their total serum IgE levels. Overall, basophils isolated from high serum IgE donors showed significantly higher levels FcεRI on their cell surface, confirming previous findings that the surface expression of this receptor correlates with soluble IgE levels in the blood^6^. Surface levels of FcεRI decreased under culture conditions in which no additional exogenous IgE was present in a time-dependent manner. The loss of receptor was reflected in the loss of surface IgE and is likely owing to the slow but steady dissociation of IgE from the receptor under these culture conditions. Addition of exogenous IgE decreased the receptor loss in basophils of high serum IgE donors and stabilized FcεRI levels in basophils of low serum IgE donors. These data provide compelling evidence, that the rate of receptor loss is higher than the rate of constitutive new receptor production in basophils from high serum IgE donors. On the other hand, this indicates that the rate of new receptor production is equal to the rate of receptor loss in basophils from low serum IgE donors. The discrepancy between the increased IgE and unaltered FcεRI levels on the surface of basophils from low serum IgE donors that were incubated with exogenous IgE is most likely owing to the fact that the anti-FcεRI-staining antibody used in this experiment shows minor steric overlap with IgE binding sites (data not shown). Collectively, our findings support the notion that IgE does not directly regulate FcεRI expression but rather prevents FcεRI internalization by stabilizing it through binding on the cell surface^31^. The exact molecular mechanism of receptor stabilization, however, is still unknown and needs further investigation.

Interestingly, we identified IL-3 as a new transcriptional regulator of FcεRI expression on human primary basophils. Incubation of basophils with IL-3 resulted in a rapid accumulation of surface IgE receptor within 24 h. The receptor increase was associated with a transcriptional upregulation of FcεRIβ, which has previously been described to promote the maturation and surface transport of hetero-tetrameric FcεRI complexes^2,39,40^. We further detected a moderate increase of FcεRIγ expression. Surprisingly, the transcription levels for FcεRIα upon IL-3 incubation decreased, emphasizing the pivotal role of the β- and γ-chain in FcεRI surface expression on allergic effector cells. Nevertheless, it is important to note that of all FcεRI subunits the α-chain showed the highest baseline expression of transcripts. Thus, our findings support previous data reporting that pre-synthesized FcεRIα can be stored intracellularly in the endoplasmatic reticulum until it associates with the homodimeric FcεRIγ alone or the FcεRIβ together with the homodimeric FcεRIγ to be transported to the cell surface via the Golgi compartment^2,41^.

In contrast to various studies using mast cells^14,15,17^ we did not observe any pro-survival effect of monomeric IgE binding on primary human basophils. Furthermore, there was no difference in blood basophil counts between low and high serum IgE donors. Nevertheless, it has previously been reported that pathologically high levels of IgE correlate with increased numbers of circulating basophils in patients with hyperimmunoglobulinemia E syndrome or in germ-free mice^34^. However, as suggested in their study the accumulation of basophils is due to an IgE-mediated upregulation of the IL-3 receptor on basophil progenitor cells in the bone marrow, which leads to increased proliferation and differentiation of the cells. Here, we focused on fully differentiated peripheral basophils, for which we could not detect any correlation between donor serum IgE levels and cell surface IL-3 receptor expression (data not shown).

Taken together, our data provide strong evidence that IL-3 signaling but not monomeric IgE-binding regulates FcεRI levels and cell survival of primary human basophils. As IL-3-mediated receptor upregulation correlated with initial FcεRI levels of the individual donors, we assume that genetic and environmental factors during basophil hematopoiesis have an additional role in the regulation of FcεRI surface levels. Overall our data suggest that targeted blocking of IL-3 signaling on human blood basophils might represent a promising strategy to prevent or treat allergic inflammation.

## Materials and Methods

### Human samples and animals

Human primary basophils were isolated from whole blood of allergic and healthy volunteers with approval from the local ethics committee. Informed consent was obtained from all donors in accordance with the Helsinki Declaration. huFcεRIα^tg^ and huFcεRIα^−/−^ on a mixed C57BL/6 J - C57BL/6 N background were obtained from Prof J.-P. Kinet^32^. Wildtype mice on a C57BL/6 J background were purchased from Janvier Labs (Saint-Berthevin Cedex, France). All animal experimentation was approved from cantonal committee on animal experimentation (BE48/15).

### Reagents

Human Sus11-IgE (NBS-C BioScience, Vienna Austria) and NIP-specific human JW8-IgE (NBS-C BioScience, Vienna Austria) were used. Both IgE clones are monomeric as assessed by the provider (Fig. S2). Recombinant human and murine IL-3 was purchased from Peprotech (London, UK). Experiments on FcεRI upregulation were performed with IL-5 (R&D Systems, Minneapolis, MN, USA) and GM-CSF (Sandoz, Basel, Switzerland). IgE and FcεRI surface levels were monitored with monoclonal mouse antibodies anti-IgE (clone IgE21) and anti-FcεRIα (clone AER-37), purchased from eBioscience (Santa Clara, USA). IL-3 (CD123) receptor and CCR3 (CD193) were detected with anti-CD123α (clone 9F5, BD Biosciences Pharmingen, San Diego, CA, USA) and anti-CCR3 (clone 5E8, BD Biosciences Pharmingen, San Diego, CA, USA) antibodies. The following antibodies were used for western blot experiments: anti-Caspase-3 (Cell Signaling Technology, Cambridge, UK), anti-Bcl-2 (DakoCytomation, Glostrup, DK), anti-GAPDH (Merck Millipore, Billerica, USA) and anti-β-actin (AC-15, Sigma-Aldrich, St. Louis, USA). Secondary HRP-labeled goat anti-rabbit and goat anti-mouse antibodies were purchased from Bio-Rad (Hercules, USA). Immunoreactive bands were visualized by enhanced chemiluminescence ECL-Kit (GE Healthcare, Chalfont St Giles, UK). Total IgE levels of volunteering blood donors were determined by ImmunoCAP measurement (Phadia AB, Uppsala, Sweden). Basophil survival was measured with Propidium iodide (100 μg/ml) purchased from Milteny Biotec GmbH (Bergisch Gladbach, Germany) and Annexin V from Biolegend (San Diego, USA). Basophil activation upon antigen challenge was determined using the FlowCAST kit from Bühlmann Laboratories AG (Schoenenbuch, Switzerland). The disruptive anti-IgE DARPin^®^ protein bi53_79 was previously characterized^29^. DARPin molecules were expressed in *E.coli* and purified from cell lysates with metal-affinity chromatography according to the manufacturer’s manual (TALON^TM^ Metal Affinity Resins User Manual, Clontech, CA). The proteins were further purified by size exclusion chromatography with a HiLoadTM 26/60 Superdex-75 prep grade column (GE Healthcare, Chalfont St. Giles, UK).

### Basophil isolation and culture

The threshold of 32 kU/L serum IgE to separate between low and high serum IgE donors was chosen arbitrarily. Human primary basophils were isolated from whole blood as previously described^35^. Dextran-sedimented supernatants from voluntary blood donors were used for Percoll density centrifugation and purified with basophil isolation kit II (Miltenyi Biotec, Bergisch Gladbach, Germany). Purity of the cells was assessed by flow cytometry using anti-CD123 and anti-CD193 antibodies. Flow cytometry data were analyzed with FlowJo (TreeStar Inc., USA). Purified basophils were cultured in 96-well U-bottom plates in complete Roswell Park Memorial Institute medium (RPMI 1640 with 10% heat-inactivated FCS, 10 mM 4-(2-hydroxyethyl)-1-piperazineethanesulfonic acid (HEPES), 100 μg/ml streptomycin, and 100 U/ml penicillin) at 37 °C, 5% CO_2_.

### Basophil desensitization and receptor kinetics

For desensitization experiments, 2.5–5 × 10^4^ isolated human primary basophils from low ( ≤ 32 kU/L) and high serum IgE donors ( > 32 kU/L) were incubated in complete RPMI medium and IgE was removed by incubation with 5 μM bi53_79 overnight. Cells were washed 3 × times with 200 μl PBS to remove dissociated IgE and DARPin^®^ proteins from the supernatant. 50 µl RPMI containing 10 ng/ml IL-3 was used to culture the cells. IgE resensitization and IgE saturation was achieved upon addition of 100 nM human Sus11-IgE for at least 60 minutes. Surface levels of IgE and FcεRI were measured every 24 h using anti-IgE and anti-FcεRI antibodies by flow cytometry.

### Basophil survival

In total, 2.5–5 × 10^4^ isolated human primary basophils from low ( ≤ 32 kU/L) and high serum IgE donors ( > 32 kU/L) were incubated overnight in complete RPMI medium in the presence or absence of bi53_79 (5 µM). IgE removal was determined by flow cytometry. The cells were incubated in medium alone and resensitized or saturated with Sus11-IgE. Basophil survival was monitored with Annexin V and popidium iodide (PI) staining and measured every 24 h. Viable cells were identified as Annexin V/PI double negative cells.

A total of 1 × 10^5^ cells from three different donors were treated under the same conditions as described before to perform western blot analysis. Proteins from every individual donor were precipitated using 10% trichloroacetic acid, pooled and separated on a NuPAGE 12% Bis-Tris gels (Thermo Fisher) and transferred to a polyvinylidene difluoride membrane (Invitrogen, USA).

### In vivo basophil quantification

Dextran-sedimented supernatants from voluntary blood donors were used for Percoll density centrifugation. The basophil cell layer was collected and cells were stained with anti-CD123 and anti-CD193 antibodies. Addition of CountBright beads (LifeTechnologies, Carlsbad, USA) allowed us to quantify the basophil (CD123^+^, CD193^+^) cell number by flow cytometry.

Blood from huFcεRIα^tg^ and huFcεRIα^−/−^ mice was collected to determine the effect of receptor expression on the absolute cell number. Basophils were identified as CD49b^+^, CD200R3^+^, and CD4^−^ cells using anti-murine CD49b (eBioscience, Santa Clara, USA), anti-murine CD200R3 (AbD Serotec, Hercules, USA), and anti-murine CD4 (Biolegend, San Diego, USA). Data from flow cytometric measurement were further processed using the FLOWJO software (TreeStar Inc., USA) and normalized to total cell number per ml blood.

### IL-3 induced upregulation of FcεRI

A total of 2.5 × 10^4^ isolated human basophils were incubated 24 h. The cells were cultured in presence and absence of IL-3, IL-5, GM-CSF, and recombinant IL-3 receptor to block IL-3 binding to CD123. In addition, IgE was removed from the cells o/n with bi53_79 and incubated with or without Sus11-IgE (100 nM) and IL-3, IL-5, GM-CSF, and recombinant IL-3. FcεRI expression and receptor-bound IgE was monitored every 6 h with Flow cytometry, as previously described. Results were normalized to the geometric mean fluorescence measured at T0 untreated. As the signal for the FcεRI receptor slightly increase upon removal of the IgE, we suggest that the staining antibody sterically interferes with receptor-bound IgE. Results were thereby corrected by subtracting a mean staining artifact (MSA). The artifact was defined as the difference in geometric mean between the untreated and the bi53_79-treated group. The artifact of every measurement was added and divided throughout the total amount of measurements to generate the MSA.

### Gene expression analysis

Human primary basophils were purified from whole blood of six donors. Total RNA was isolated using the RNeasy Mini kit (QIAgen) following manufacturer instructions. The isolated amount of RNA was quantified on a NanoDrop (Thermo Scientific) and tested for quality using a bioanalyzer (Agilent). RNA fulfilling quality criteria (RIN ≥ 6) of basophils from three different donors were pooled and used for whole-genome array (Agilent 44k) measurements. Two individual arrays were performed for each condition. Data were analyzed and mined using the R project for statistical computing. Measured spot intensities were normalized within and across arrays. Values were log2-transformed. For several genes of interest the relative log2 fold change (RLFC) between medium treated cells and IL-3-treated cells was calculated.

### IL-3 upregulated FcεRI functionally activates basophils

Purified human blood basophils were seeded at 2.5 × 10^4^ cells per well in medium in the presence and absence of IL-3 for 18 h. The cells were then sensitized with NIP-specific JW8-IgE for 30 min at 37 °C and washed twice with 150 µl of PBS. For activation experiments, sensitized cells were challenged with NIP-(7)-BSA (0.01-100 nM) from Biosearch Technology (Petaluma, USA) or anti-FcεRI from Bühlmann Laboratories for 25 min at 37 °C. Basophil activation was determined by measuring surface expression of basophil activation marker CD63 with FlowCAST from Bühlmann Laboratories. IL-3-mediated FcεRI upregulation correlation was performed comparing initial FcεRI level with IL-3-treated FcεRI level. Total surface IgE upon endogenous JW8-IgE sensitization of IL-3- and non-IL-3-treated basophils was compared.

### Generation of huFcεRIα^tg^ BMMCs

Femors and tibiae of a huFcεRIα^tg^ mouse were flushed with complete RPMI medium. The isolated bone marrow was filtered through a 70 μm cell strainer. Subsequently, the cells were washed, resuspended in 25 ml BMMC culture medium (complete RPMI supplemented with sodium pyruvate, L-glutamine, non-essential amino acids, β-mercaptoethanol and recombinant murine IL-3) and seeded in a T75 flask. The cells were cultured for 4 weeks at 37 °C, 5% CO_2_ and medium was changed twice a week.

### Statistics

Statistical analysis was carried out with Prism 6.0 software (GraphPad Software, La Jolla, Calif). All data are shown as means ± SEM. Comparisons between different treatments were analyzed by using Student’s *t*-test or one-way analysis of variance. *P* values of < 0.05 were considered statistically significant.

## Electronic supplementary material

Supplementary figures

Supplementary Information
