# Supplementary figures and images for: IL-3 but not monomeric IgE regulates FcεRI levels and cell survival in primary human basophils
Source: Cell Death Dis. 2018 May 3;9(5):510. doi: 10.1038/s41419-018-0526-9 (PMC5938712; doi:10.1038/s41419-018-0526-9)

Supplementary Figure 1

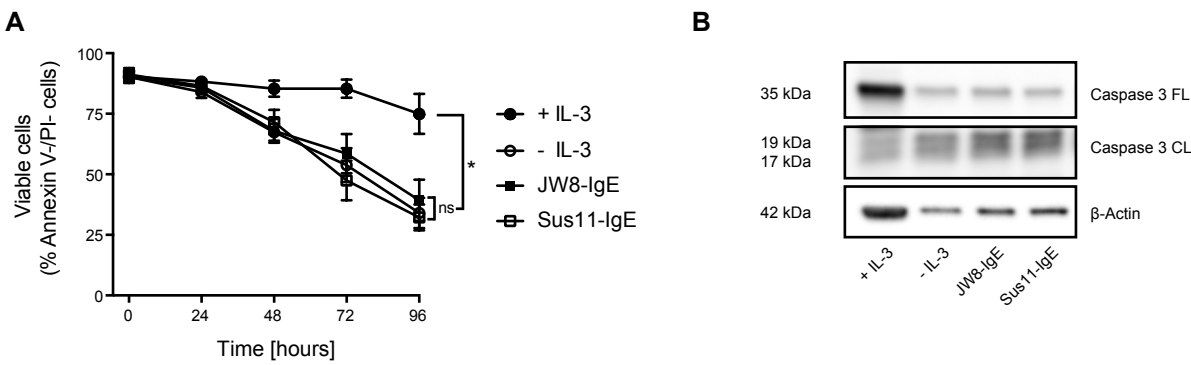

Supplementary Figure 2

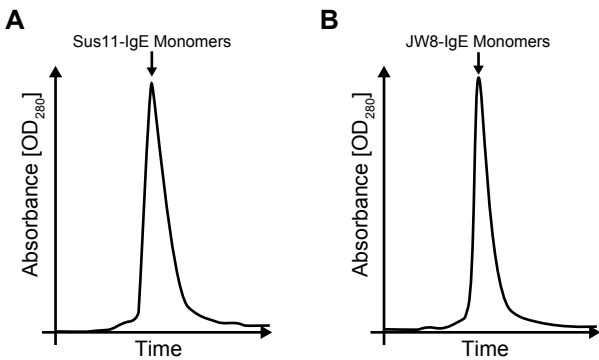

Supplement: Supplementary file 1 — Supplementary figures [file 41419_2018_526_MOESM1_ESM.pdf]
