# Supplementary material for: IL-3 but not monomeric IgE regulates FcεRI levels and cell survival in primary human basophils
Source: Cell Death Dis. 2018 May 3;9(5):510. doi: 10.1038/s41419-018-0526-9 (PMC5938712; doi:10.1038/s41419-018-0526-9)
Supplement: Supplementary file 2 — Supplementary Information [file 41419_2018_526_MOESM2_ESM.docx]

**Supplementary Information: IL-3 but not monomeric IgE regulates FcεRI levels and cell survival in primary human basophils**

Fabian Zellweger^1,2*^, Patrick Buschor^1,2*^, Gabriel Hobi^1,2^, Daniel Brigger^1,2^, Clemens A. Dahinden^3^, Peter M. Villiger^1,2^, Alexander Eggel^1,2**^

^1^Department of BioMedical Research, University of Bern, Bern, Switzerland

^2^Department of Rheumatology, Immunology and Allergology, University Hospital Bern, Bern, Switzerland

^3^former Institute of Immunology, University of Bern, Bern, Switzerland

*These authors contributed equally to this work.

**Corresponding author: Alexander Eggel, PhD, University of Bern, Department of Rheumatology, Immunology and Allergology, Sahlihaus 2, Bern, Switzerland; Phone: +41 31 632 22 87; Fax: +41 31 381 57 35; Email: alexander.eggel@dbmr.unibe.ch

**Supplementary Fig. 1. *Survival of BMMCs expressing the human FcRεIα in the presence or absence of monomeric IgE.*** BMMCs from a huFcεRIα^tg^ mouse were incubated with murine IL-3 (30 ng/ml), no IL-3, monomeric Sus11-IgE or monomeric JW8-IgE (100 nM). (**A**) Cell viability was assessed by flow cytometry over a 96-hour time period. (**B**) Comparative western blot analysis of full length and cleaved caspase-3 in huFcεRIα^tg^ BMMCs that were cultured for 96 hours. β-actin serves as loading control. Statistical significance was calculated by one-way ANOVA at 96 hours and data are shown as mean ± SEM, n = 4.

**Supplementary Fig. 2. *Assessment of protein aggregation.*** Gel permeation chromatography of Sus11-IgE (A) and JW8-IgE (B) on a superose12/HR column.
